# Livedoid vasculopathy, calciphylaxis, and Martorell’s hypertensive ulcer: update on ischemic ulcers due to impaired microcirculation of the lower limbs^☆^

**DOI:** 10.1016/j.abd.2024.09.004

**Published:** 2025-01-22

**Authors:** Priscila Neri Lacerda, Lucas Campos Garcia, Izabelle Ferreira da Silva Mazeto, Hélio Amante Miot, Luciana Patricia Fernandes Abbade

**Affiliations:** a Santa Casa de Misericórdia da Bahia, Hospital Santa Izabel, Salvador, BA, Brazil; b Hospital das Clínicas, Faculty of Medicine, Universidade Federal de Minas Gerais, Belo Horizonte, MG, Brazil; c Department of Infectology, Dermatology, Diagnostic Imaging and Radiotherapy, Faculty of Medicine, Universidade Estadual Paulista, Botucatu, SP, Brazil

**Keywords:** Calcific uremic arteriolopathy, Calciphylaxis, Hypertension, Livedoid vasculopathy

## Abstract

Ischemic ulcers due to compromised microcirculation of the lower limbs cause painful ulcers that represent a challenge for the correct diagnosis and treatment. Livedoid vasculopathy, calciphylaxis, and Martorell’s hypertensive ischemic ulcer are part of this group and present some similarities due to microvascular occlusive impairment. They are often misdiagnosed as inflammatory ulcers such as pyoderma gangrenosum and vasculitis. This review discusses the pathophysiology, risk factors, clinical aspects, differential diagnoses, histopathology, and presents a therapeutic update of livedoid vasculopathy, calciphylaxis, and Martorell’s ulcer. Although they are less frequent causes of chronic ulcers, a correct diagnosis is essential to reduce the chance of erroneous therapies that may impact morbidity and mortality related to these conditions.

## Introduction

Chronic lower limb ulcers are common conditions among adults, with several causes, and often represent a diagnostic and therapeutic challenge. The main causes are venous, arterial, and neuropathic diseases.^1^ Ischemic ulcers, such as those of arterial origin, develop due to inadequate blood supply caused by arterial macrocircluation impairment. Their most common cause is atherosclerotic disease.^2^ However, this paper addresses other causes of ischemic ulcers of the lower limbs, related to occlusive impairment of the microcirculation, such as livedoid vasculopathy, calciphylaxis, and hypertensive ischemic ulcer. Although they are less common causes of chronic lower limb ulcers, they should be recognized clinically and be part of the differential diagnoses, as they are often misdiagnosed, which results in diagnostic and treatment delays.^3^

## Livedoid vasculopathy

### Definition

**Fig. 1:**
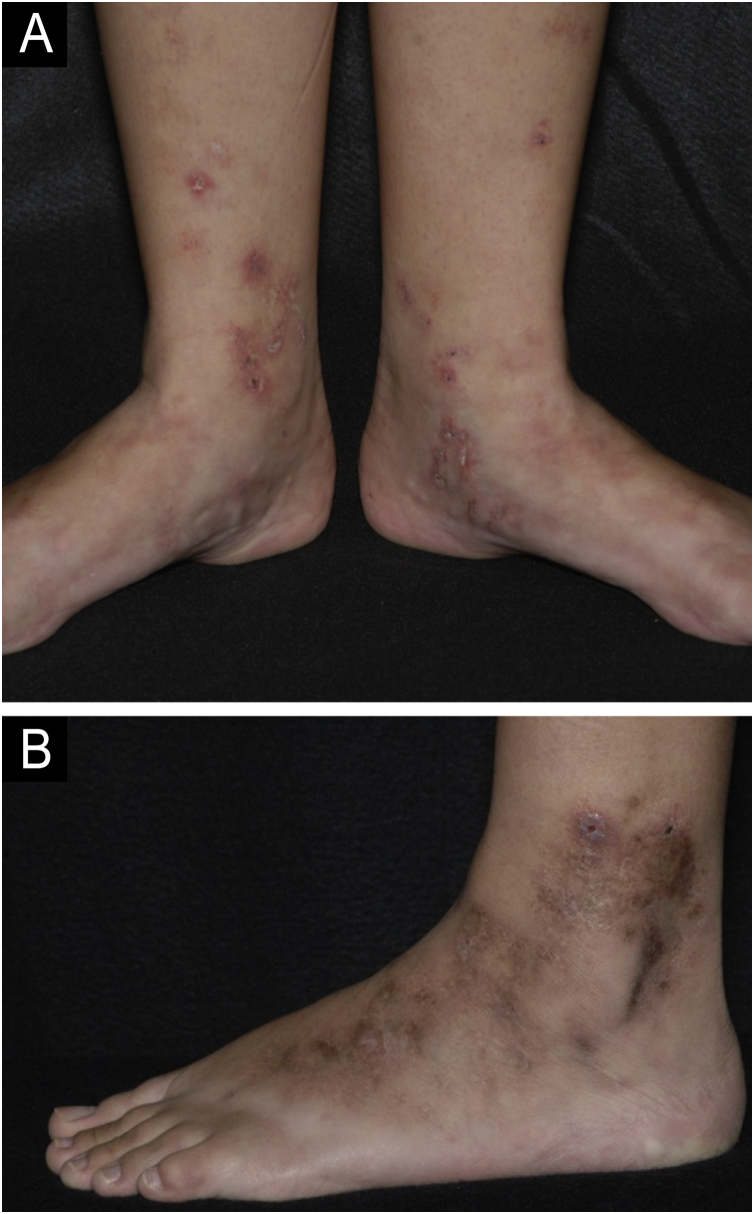
Livedoid vasculopathy. (A) Active lesions characterized by small ulcers with crusts and erythematous-purpuric edges located on the medial side of the legs and feet. Livedo racemosa is also noted on the dorsum of the feet; (B) Lesions in remission on the lateral side of the leg and foot with brownish scars and ivory-white stellate scars characterizing Millian's white atrophy.

Livedoid vasculopathy (LV) is a chronic ulcerative disease with a clinical course characterized by periods of remissions and exacerbations. It affects the distal region of the legs, ankles and dorsum of the feet (Fig. 1).^4^ The condition is intensely painful and causes a significant reduction in the quality of life.^5^ It is a thrombotic disease distinct from vasculitis because it is not primarily characterized by damage to the vessel wall or inflammatory infiltrate. Some authors do not consider it a single entity, but rather the cutaneous manifestation of different diseases that present hypercoagulability, thrombosis of the dermal vessels and fibrinolysis reduction, such as autoimmune diseases, neoplastic syndromes, congenital or acquired anomalies of the fibrinolytic system, among others.^6^, ^7^

### Epidemiology and risk factors

LV is considered a rare disease with an estimated incidence of 1:100,000 per year. It predominates in women at a ratio of 3:1 and the lesions may worsen in the hottest months of the year.^7^ The most affected age group is 15 to 50 years, with a mean age of 32 years.^5^

Hereditary and acquired mutations that predispose to hypercoagulability have been identified. The most frequently involved genes are MTHFR (methylenetetrahydrofolate reductase), PAI-1 (plasminogen activator inhibitor-1), prothrombin and factor V gene.^8^ A systematic review found the PAI-1 ‒ 675 4 G/5G mutation as the most frequent (85.26%), while PAI-1 A844 G, MTHFR C677 T and MTHFR A1298C followed in order of prevalence, respectively. In patients from Europe, North America and South America, the G20210A mutations in the prothrombin gene and G1691A in the factor V gene were also found in significant numbers of patients.^8^

Systemic prothrombotic conditions are considered the main risk factors, such as antiphospholipid antibody syndrome (APAS), collagen diseases (systemic lupus, rheumatoid arthritis, Sjögren's syndrome, mixed connective tissue disease) and sickle cell anemia.^9^ Statistically the most often associated comorbidities are diabetes, systemic arterial hypertension and peripheral venous disease.^10^ Table 1 highlights the main comorbidities associated with LV.

**Table 1:** Comorbidities associated with livedoid vasculopathy.

Comorbidities	
Metabolic	Diabetes mellitus, familial hypertriglyceridemia, hyperthyroidism, obesity, hyperhomocysteinemia
Cardiocirculatory	Systemic arterial hypertension, peripheral venous insufficiency, coronary artery disease
Rheumatological	Rheumatoid arthritis, systemic lupus erythematosus, antiphospholipid antibody syndrome, mixed connective tissue disease
Hematological	Systemic lymphomas, monoclonal gammopathies, polycythemia vera, acquired thrombophilias (cryoglobulins, cryoagglutinins, cryofibrinogen), congenital thrombophilias (associated with mutations in factors V, VIII, IX, prothrombin gene, proteins C and S, antithrombin III, plasminogen factor inhibitor and methylenetetrahydrofolate reductase [MTHFR] gene)
Infectious	HIV, hepatitis B and C
Miscellaneous	Breast cancer, asthma, migraine, kidney transplant, scleroderma

### Pathophysiology

Although the pathophysiological process has not yet been fully elucidated, non-inflammatory thrombosis of the dermal vessels seems to be a key event. The reduction in blood flow velocity, endothelial damage and changes in coagulation factors favor hypercoagulability and act synergistically to trigger disease onset. The thrombotic process may extend to the vasa nervorum and cause neuropathy.^7^ At the molecular level, the increase in prothrombotic factors such as factors VIII, IX, antiphospholipid antibodies, homocysteine ​​and lipoprotein(a) are the most frequently reported alterations.^6^, ^10^, ^11^ Criado et al. in a study of 75 Brazilian patients with LV, found that approximately 66% of the cases had thrombophilic factors, with lipoprotein(a) as the most common factor detected.^10^

Skin lesions occur due to pericapillary deposition of fibrin and the formation of thrombi that act as a barrier to oxygen diffusion to the tissues, causing ischemia and low tissue perfusion that impairs healing. Moreover, hypoxia makes the affected area an ineffective barrier to bacteria, increasing the risk of infection.^12^

### Clinical manifestations

The classic manifestation includes small, deep, necrotic, and painful ulcers that affect the distal region of the legs, especially the malleolar region and dorsum of the foot (Fig. 1A). The ulcers heal slowly and leave characteristic atrophic ivory-white scars known as Millian's white atrophy (Fig. 1B). Livedo racemosa may be observed in these patients, comprising the classic triad (Fig. 1A).^6^, ^7^ Livedo reticularis can also be found, both in the lower limbs and, more rarely, in other parts of the body, such as the hands, hips, and abdomen.^4^

The ulcers may be preceded by purpuric macules and papules. At this early stage, intense and debilitating pain may already be evident.^4^ Pain may be aggravated by the presence of neuropathy (in more than 10% of patients), which should be actively investigated, particularly in cases of refractory pain. The mononeuritis multiplex pattern is the most prevalent in electroneuromyography, determining asymmetrical painful sensations that are not restricted to the ulcerated areas.^13^ Although it occurs less frequently, pruritus may also be present.^4^

In the natural history of LV, lesions recur and resolve without a clear trigger, and it is common for the patient to have multiple lesions in different stages of healing.^14^

As previously mentioned, LV may be associated with hypercoagulable states, autoimmune diseases, and venous stasis; however, more than half of the cases occur in individuals without comorbidities.^14^ It is recommended that in addition to a complete physical examination, laboratory evaluation be requested to investigate possible associated systemic causes: thrombophilia, antiphospholipid antibodies, presence of paraproteins, screening for collagenosis, and evaluation of liver and kidney functions (Table 1).^15^

There is a recent proposal to classify LV according to the clinical and laboratory alterations found: 1) Idiopathic (without underlying clinical and laboratory alterations); 2) Associated with hypercoagulable states; 3) Associated with rheumatological disease; 4) With concomitant venous disease; 5) Associated with other mechanisms or multifactorial mechanisms.^14^

### Histopathology

**Fig. 2:**
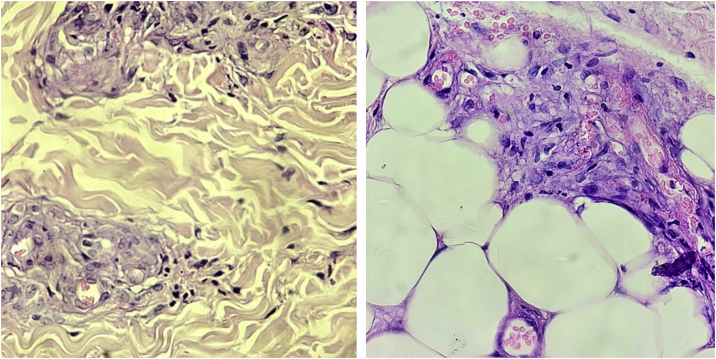
Histopathology of livedoid vasculopathy. Vessel proliferation in the superficial and deep dermis, with vessel wall thickening. Small foci of necrosis and small thrombi in the superficial vessels (Hematoxylin & eosin, ×1000).

Histopathological examination is useful for diagnostic confirmation. In some cases, more than one biopsy may be necessary.^10^, ^15^ The classic findings are thrombi in the dermal vessels and sparse perivascular lymphocytic inflammatory infiltrate. Deposition of fibrinoid material in the vessel walls and in the dermis may also be observed (Fig. 2).^15^

The presence of a neutrophilic infiltrate is unusual, but it may be present in biopsies performed in old, ulcerated areas.^10^ The identification of leukocytoclasia suggests vasculitis should be considered in the differential diagnosis.^15^

Direct immunofluorescence is positive in most cases.^10^, ^16^ The deposition occurs mainly in the blood vessels, but can also be seen at the dermoepidermal junction. IgM and C3 deposition are the most common, but IgG and IgA deposition may also occur.^15^ Biopsies should be performed in the most recent lesions, preferably without ulceration.

### Differential diagnosis

The differential diagnosis includes inflammatory and vaso-occlusive diseases that cause ulcerations in the lower limbs. Among the inflammatory diseases, pyoderma gangrenosum and medium-caliber vasculitis (polyarteritis nodosa (PAN) and antineutrophil cytoplasmic antibody (ANCA)-associated vasculitis) stand out.^15^ Among the vaso-occlusive disorders, calciphylaxis, Martorell’s hypertensive ulcer, endocarditis, Lucio phenomenon, cholesterol embolism, cryoglobulinemia and ulcers induced by cocaine contaminated with levamisole should be ruled out.^15^

Histopathology, associated with the clinical manifestations and a complementary laboratory investigation are necessary for diagnostic definition. In cases where neuropathy is present, the differential diagnosis with PAN must be well established and, therefore, biopsies must reach the deepest portions of the subcutaneous tissue to represent medium-sized vessels.^13^

### Treatment

The treatment of LV is challenging and generally requires a combination of therapeutic options with distinct and synergistic mechanisms of action (Table 2). According to a systematic review, most studies classify the therapeutic response as partial, when there is some incomplete improvement, remission, that is, healing of all lesions, and recurrence when there is a new outbreak. According to this systematic review, approximately 44% of the patients do not respond or do not tolerate the first therapeutic options.^5^

**Table 2:** Main drug treatments for livedoid vasculopathy.

Class	Medication	Dose	Main adverse events
**Antiplatelet agents**^a^**(First line of treatment)**	Acetylsalicylic Acid	75‒325 mg/day	Bleeding (gastric mucosa), dyspepsia, dizziness, urticaria, angioedema
	Clopidogrel	75 mg/day	Bleeding, dyspepsia, diarrhea
	Pentoxifylline	400 mg 3x/day	Headache, gastrointestinal discomfort, facial flushing, risky in patients with arrhythmias
**Anticoagulants**^a^**(First line of treatment)**	Apixaban	2,5 mg 2x/day	Bleeding, dyspnea, nausea, increased transaminases and thrombocytosis
	Rivaroxaban	20 mg 1x/day	
	Dabigatran	150 mg 2x/day	Bleeding, dyspepsia, nausea
	Warfarin	Maintain INR between 2‒3	Bleeding, fever, nausea, vomiting
	Low molecular weight heparins	Enoxaparin 1 mg/kg SC 1x or 2x/day	Bleeding, confusion, nausea, anaphylactoid reactions
**Systemic steroids (Second line of treatment)**	Prednisone	0.5 to 1.0 mg/kg/day	Hypertension, hyperglycemia, infections, insomnia, irritability
	Danazol	200 mg/day	Menstrual irregularity, female virilization, worsening of prostatic hyperplasia, petechiae, hepatotoxicity
**Peripheral vasodilators (Second line of treatment)**	Cilostazol	50‒100 mg 2x/day	Tachycardia, headache, diarrhea, peripheral edema
	Nifedipine	20 mg 3x/day	Myalgia, peripheral edema, constipation, abdominal pain
**Classical Immunosuppressants (Third line of treatment)**	Ciclosporin	3‒5 mg/kg/day	Hypertension, nephrotoxicity, opportunistic infections, cytopenias, malignant neoplasms
	Azathioprine	2‒3 mg/kg/day	Gastrointestinal, opportunistic infections and cytopenias
	Sulfasalazine	2‒3 g/day divided in three doses	Gastrointestinal intolerance, headache, agranulocytosis, hepatotoxicity
**Immunomodulators/miscellaneous (Third line of treatment)**	Hydroxychloroquine	4‒6 mg/kg/day	Retinopathy, nausea, vomiting, skin pigmentation, dizziness, headache, ototoxicity and peripheral neuropathy
	Dapsone	50‒200 mg/day	DRESS syndrome (drug reaction with eosinophilia and systemic symptoms), methemoglobinemia, and agranulocytosis
	Colchicine	0.5 mg 2 to 3x/day	Diarrhea, vomiting, colic, headache, asthenia
	Doxycycline	100‒200 mg/day	Dyspepsia, teeth pigmentation, loss of appetite
	Intravenous immunoglobulin (in refractory cases)	2 g/kg divided in three to five days every four weeks	Headache, nausea, vomiting, fatigue

a Antiplatelet agents and anticoagulants can be used together.

Non-pharmacological treatment involves measures aimed at reducing venous stasis and stopping smoking.^7^ Compression therapies should be prescribed as soon as peripheral arterial disease is excluded, by palpation of peripheral pulses, ankle-brachial index measurement or imaging tests, such as arterial Doppler of the lower limbs.^15^ Comorbidities such as heart disease and diabetes must be optimally controlled.^6^, ^17^ Patients with hyperhomocysteinemia can be treated with folic acid, vitamin B6 and B12 replacement.^7^

Antiplatelet agents are considered the first step in the treatment escalation. They act by preventing thrombus formation through vasodilation and cyclooxygenase inhibition.^5^ Acetylsalicylic acid (ASA) should be used at a dose of 75 to 325 mg/day, ^7^ and clopidogrel at a dose of 75 mg/day is also an option.^18^ Pentoxifylline is a xanthine with antiplatelet and vasodilatory properties, and additionally increases the flexibility of leukocytes and erythrocytes, allowing a better flow. It is used alone or in combination with other antiplatelet agents. Severe heart disease and hypotension are contraindications. The usual dose is 400 mg two to three times a day.^7^ Gastrointestinal side effects may limit the use of pentoxifylline and ASA.^10^

Anticoagulants are the drugs with the best response as monotherapy and, therefore, they can be considered as first-line treatment along with antiplatelet agents.^5^, ^17^ The new oral anticoagulants that act as selective inhibitors of coagulation factor Xa (apixaban, edoxaban and rivaroxaban) or prothrombin (dabigatran) are an excellent therapeutic option. Lower doses than usual can be effective. ^19^ This class of drugs has a rapid onset of action and does not require serial laboratory measurements.^7^ Treatment can be continuous or intermittent in less severe cases.^20^ Despite the practical and effective use of the new oral anticoagulants, warfarin and low molecular weight heparins are still a therapeutic option for patients with refractory activity.^6^

Systemic steroids, such as prednisolone and danazol, are an adjuvant therapeutic option and can be used in combination with antiplatelet agents and anticoagulants. They are more effective when patients have rheumatological comorbidities, such as rheumatoid arthritis and systemic lupus erythematosus. In addition to their anti-inflammatory effects, steroids act by accelerating fibrinolysis and inhibiting coagulation.^5^ Danazol accelerates the hepatic production of proteins C and S, and is also especially useful in patients with increased lipoprotein(a). It is generally well tolerated, but up to one-fifth of patients may experience some adverse event, such as increased prostatic hyperplasia, menstrual irregularity, virilization in female patients, and liver alterations, which should be monitored.^5^, ^10^

Classic immunosuppressants and drugs with anti-inflammatory action, such as hydroxychloroquine, colchicine, dapsone, doxycycline, azathioprine, cyclosporine and sulfasalazine, may be associated in the treatment. They are usually prescribed together with antiplatelet agents and/or anticoagulants.^5^, ^15^

Peripheral vasodilator drugs such as nifedipine and cilostazol may improve pain and aid healing.^7^ Still in this line of action, aiming at the sympathetic vasoconstriction inhibition, the application of perilesional botulinum toxin A was recently reported presenting good results, but with lesion recurrence in approximately three months.^21^

Given the number of therapeutic options and management difficulty, a group of authors attempted to rationalize the initial combined management through an approach called CHAP (Calcium channel blockers, Hydroxychloroquine, Acetylsalicylic acid and Pentoxifylline), with a total response rate of over 80% in the two-year follow-up, but the small sample size of this study with only 12 patients is noteworthy.^22^

Intravenous immunoglobulin (IVIG) is a rescue therapy reserved for cases refractory to previous treatments.^5^, ^7^ Although the exact mechanism of action is not well understood, inhibition of antiphospholipid antibody production, platelet aggregation, and endothelial dysfunction could explain the clinical response.^5^ A systematic review found a 95% response rate with doses of 1–2.1 g/kg, divided into two to three consecutive days and repeated every four weeks. The treatment interval was increased and the medication was discontinued according to the attending physician experience. The first sign of clinical response was pain improvement, but the ulcers took days to weeks to heal. Recurrence occurred in approximately one-third of patients.^23^ Cases that do not respond to IVIG can be treated with tissue plasminogen activator factor, but its use requires a hospital environment and strict control of hemorrhagic complications.^7^

Hyperbaric oxygen therapy is an adjuvant modality, limited by availability and costs. It is based on the plasma dissolution of oxygen promoted by the supply of 100% oxygen under high pressure (two to three times the atmospheric pressure), promoting hypoxic tissue oxygenation.^5^

The use of anti-TNFα immunobiologicals and JAK inhibitors has been recently evaluated for refractory cases, rapidly progressive cases or those with contraindications to the use of antiplatelet agents and anticoagulants. However, this use is still incipient and further studies are required.^15^, ^23^, ^24^, ^25^, ^26^

Therefore, the treatment of LV remains a challenge with several therapeutic options and variable healing rates. Furthermore, most treatments are based on studies, mainly case reports and case series, which provide low levels of evidence.^5^

## Calciphylaxis

### Introduction

Calciphylaxis, also known as uremic calcific arteriolopathy, is a rare, cutaneous-systemic, and life-threatening vasculopathy. It is characterized by intense calcium deposition in small blood vessels of the deep dermis and subcutaneous tissue, usually associated with secondary hyperparathyroidism with calcium and phosphorus metabolism alteration. Patients with end-stage renal disease (low glomerular filtration rate or renal replacement therapy) represent the main population affected by the disease (uremic calciphylaxis). However, the presence of renal disease is not an absolute requirement for the diagnosis and the condition may be observed even in the absence of comorbidities; these cases are referred as non-uremic calciphylaxis. The intensely painful and necrotic skin ulcers contribute to the significant mortality rate of the disease (60%-80%), even when diagnosed in the early stages.^27^, ^28^

The term calciphylaxis was coined after Professor Hans Selye and his collaborators conducted experiments on the mechanism of metastatic calcification in 1961. They exposed rats to very high doses of vitamin D or parathyroid hormone, associated with a diet rich in phosphorus or intravenous/intraperitoneal administration of iron salts. Thus, they were able to demonstrate massive metastatic calcification of the subcutaneous tissue. In this experiment, the development of cutaneous calcification was considered an adaptive or phylactic reaction and was referred to as calciphylaxis (calcification and phylaxis).^29^, ^30^ However, this experimental model did not demonstrate atherosclerosis with medial calcification, which is currently considered the basic pathophysiology of calciphylaxis. To date, the term calciphylaxis is widely used based on this experiment, but it is inadequate to explain the disease pathophysiology.^30^, ^31^, ^32^

After this experiment, case reports were published describing patients with renal failure who developed generalized subcutaneous calcifications. The authors correlated these clinical cases with Selye's experimental model, diagnosing these patients with calciphylaxis. Several similar reports were subsequently presented using the term calciphylaxis, which is widely used to this day.^30^

### Epidemiology

The incidence of calciphylaxis in patients on renal replacement therapy ranges from 0.04% to 4%, with a growing increase in the last decade. The interval between starting dialysis and disease onset is approximately 30 months. Patients treated with peritoneal dialysis have a higher incidence when compared to those treated with hemodialysis. The mean age at diagnosis ranges from 50 to 70 years, and it is rare in children. Approximately 60% to 70% of the patients with calciphylaxis are female, and some studies report a higher incidence in Caucasians.^33^, ^35^

### Pathophysiology and risk factors

**Fig. 3:**
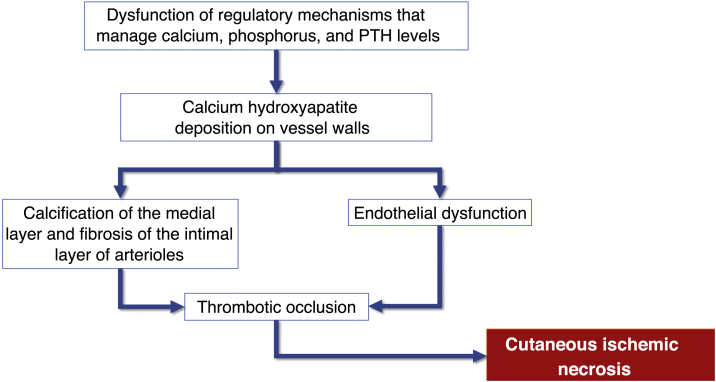
Flowchart of the sequence of events related to the pathophysiology of calciphylaxis: calcification of arterioles, endothelial lesion and consequent thrombotic occlusion of these vessels. These alterations cause ischemia and necrosis of the subcutaneous tissue, with the appearance of necrotic ulcers in the most advanced stages. PTH, Parathyroid hormone.

Although the pathogenesis of calciphylaxis involves multiple etiological factors, the complex chain of events results from inflammation and a hypercoagulable state secondary to alterations in calcium and phosphorus metabolism, with consequent vascular calcification and cutaneous ischemic necrosis (flowchart – Fig. 3). The initial event is the progressive lumen narrowing of the cutaneous and subcutaneous microcirculation produced by calcification within the medial layer (known as medial calcification), endothelial cell proliferation, and fibrosis below the intima (known as subintimal fibroplasia). Subsequently, thrombosis develops in the vessel lumen, promoting vascular occlusion and ischemic lesions.^35^

Vascular calcification is associated with the lack of molecular calcification inhibitors in the vessel wall. One of the main controllers of this mechanism is vitamin K, which activates a potent calcification inhibitor called matrix Gla protein (MGP), secreted by vascular smooth muscle cells and endothelial cells.^36^ Therefore, conditions that lead to vitamin K deficiency favor vascular calcification. In patients with end-stage renal disease, vitamin K deficiency may occur due to a lower intake of foods rich in the substance and also due to the frequent use of vitamin K-inhibiting anticoagulants, such as warfarin.^37^ According to a German study with data from a large national registry, approximately 50% of the patients diagnosed with calciphylaxis and kidney disease were using warfarin at the time of the diagnosis.^36^

Patients with calciphylaxis also have a high prevalence of congenital or acquired hypercoagulability. These conditions favor thrombosis in the microcirculation, which is the final event that causes vascular occlusion. The most common alterations are the presence of lupus anticoagulant antibody, antithrombin deficiency, and protein C and S deficiency.^38^

Several other risk factors have been identified, such as female gender, caucasian ethnicity, diabetes mellitus, obesity, prolonged dialysis time, hypoalbuminemia, and alterations in calcium and phosphorus metabolism.^27^, ^29^

### Clinical manifestations and diagnosis

**Fig. 4:**
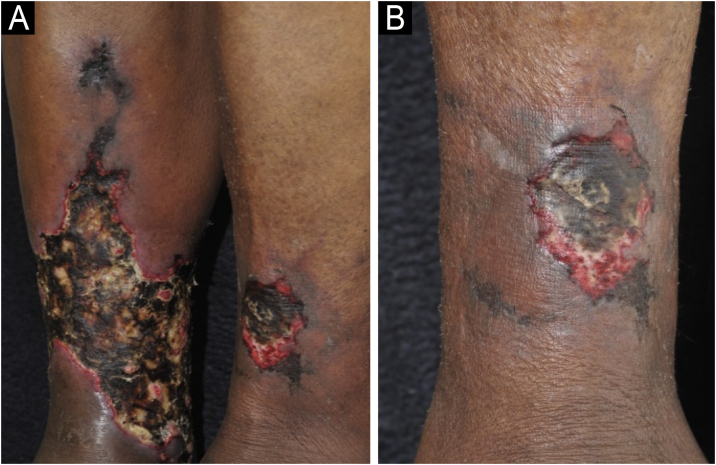
Calciphylaxis: (A) Retiform purpura and advanced stage of necrotic ulcers in the classical reticulated format in the lower limbs. (B) Close-up of the necrotic ulcer (patient of Ref. 39).

Calciphylaxis can be clinically classified as uremic when it occurs in patients with end-stage chronic renal disease, or non-uremic, when it occurs in patients with normal renal function or early stages of chronic renal disease with risk factors such as morbid obesity, arterial hypertension and type 2 diabetes mellitus as a result of a metabolic syndrome. Uremic calciphylaxis is the most common. However, the clinical manifestations are similar regardless of the association with end-stage chronic renal disease.^3^, ^34^ Calciphylaxis should be suspected in patients with the risk factors mentioned above and who initially present with painful, purpuric/erythematous nodular lesions or subcutaneous plaques associated with livedo racemosa, especially when present in areas of greater adiposity such as the trunk and lower limbs. The initial lesions rapidly progress to intensely painful necrotic ulcers, with centrifugal extension, in the classic reticulated format (Fig. 4). Secondary bacterial infection may be associated, and is one of the main causes of death.^27^, ^28^, ^30^ Although skin lesions are the main clinical signs of the disease, other organs, such as the lungs, skeletal muscles, pancreas, brain, eyes and digestive tract may also be involved.^30^

The patient history should be obtained and a complete examination performed to identify additional skin lesions. In patients receiving warfarin, a differential diagnosis between calciphylaxis and warfarin necrosis should be made. Other important differential diagnoses are livedoid vasculopathy, hypertensive ischemic ulcer, pyoderma gangrenosum, purpura fulminans and necrotizing vasculitis.^34^

Clinical suspicion is important for early diagnosis and laboratory tests can help in achieving the diagnosis. Elevations in serum calcium or phosphate levels are not always found, but when altered they corroborate the diagnosis.^39^ It is considered altered when the calcium/phosphate product is greater than 70 mg^2^/dL^2^. However, according to a German study, approximately 86% of dialysis-dependent patients with calciphylaxis had normal or low serum calcium levels and 40% had normal or low phosphate levels.^36^

**Fig. 5:**
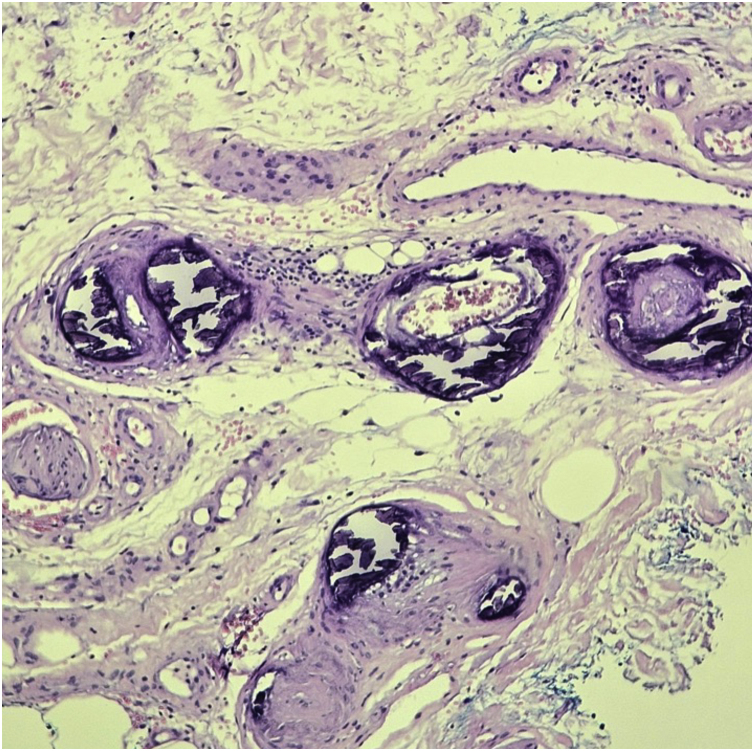
Histopathology of calciphylaxis lesion. Necrosis of subcutaneous fat, with significant calcium deposition on the vessel wall and intima proliferation (Hematoxylin & eosin, ×400).

Diagnostic confirmation is obtained with histopathology of a skin biopsy at the lesion periphery deep enough to reach the subcutaneous tissue, outside necrotic areas. The main histopathological findings are calcification of small vessels, intimal hyperplasia, and thrombotic occlusion of vessels in the dermis and subcutaneous tissue. Inflammatory infiltration is frequently observed (Fig. 5). Specific stains to demonstrate the presence of calcium in the tissue, such as von Kossa staining, are important to confirm the diagnosis.^27^, ^40^

### Treatment

The treatment of calciphylaxis is multidisciplinary and is based on risk factors control, effective pain control, local management of the lesions, including the treatment of possible secondary infections, and specific treatment with medications to stabilize the disease (Table 3).^33^

**Table 3:** Main treatments for calciphylaxis.

General measures	Details
**Risk factor control**	Discontinuation of warfarin, vitamin D and calcium phosphate
	Maintenance of serum parathyroid hormone (PTH) levels between 150‒300 ng/mL
	Cinacalcet for treatment of secondary hyperparathyroidism
	Intensification of dialysis
	Parathyroidectomy if refractory secondary hyperparathyroidism
**Pain control**	Analgesia with opioids (morphine, codeine and hydrocodone should be avoided because they have active metabolites that accumulate in renal failure and cause respiratory depression)
	Consider an evaluation from a specialist in analgesic therapy
**Local ulcer management**	Surgical debridement
	Enzymatic (collagenase or papain) or autolytic (hydrocolloid or hydrogel) debridement
	Dressings
	Skin grafts
	Hyperbaric oxygen therapy
**Secondary infection treatment**	Systemic antibiotics
**Specific treatment**	Dose: 25 g in 100 mL of solution administered intravenously three times a week during the last 30 to 60 minutes of hemodialysis
Sodium thiosulfate	Main adverse events: nausea, vomiting, metabolic acidosis and sepsis

When risk factors are controlled, warfarin (a vitamin K antagonist), vitamin D, and calcium phosphate should be discontinued. Dialysis should be intensified to control mineral metabolism to the target calcium, phosphorus, and parathyroid hormone levels. Another strategy is to replace activated vitamin D with cinacalcet to achieve target parathyroid hormone levels. Parathyroidectomy is indicated in cases of severe refractory hyperparathyroidism.^33^

The local treatment of lesions is essential to improve pain and healing. The objectives are to remove exudate and necrotic tissue, in addition to preventing infection. Surgical debridement of necrotic tissue should be performed whenever possible, despite the challenge presented by the associated intense pain. The use of other debridement methods is also indicated, such as enzymatic debriding agents (collagenase or papain) or autolytic agents (hydrocolloid or hydrogel), to avoid tissue trauma or excessive manipulation. Systemic antibiotics should be prescribed if infection is suspected. After wound stabilization with the presence of granulation tissue, dressings should be used to maintain a moisty environment or a split-thickness skin graft. There are also studies reporting the benefits of hyperbaric oxygen therapy.^34^, ^41^, ^42^

The specific treatment of calciphylaxis can be carried out with sodium thiosulfate (a vasodilator agent capable of inhibiting vascular calcification by adipocytes) and bisphosphonates (agents capable of inhibiting phosphorus transportation and reducing the formation of calcium phosphate crystals).^41^

Sodium thiosulfate is the most frequently used drug.^43^ The usual dose is 25 g in 100 mL of solution administered intravenously three times a week during the last 30 to 60 minutes of hemodialysis. The ideal duration of the treatment course is unclear. In a systematic review that included case reports and case series, totaling 358 patients with calciphylaxis and the use of sodium thiosulfate, 96.1% of the patients were on dialysis and the treatment was effective in 70.1% of the patients. The doses of calcium thiosulfate ranged from 5 to 25 grams and were administered three times a week, on average, until lesions stabilized. The administration was intravenous (70.3%), intraperitoneal (9.8%), during dialysis sessions (11.1%), and intravenous in combination with oral tablets (7.3%). There was no significant difference in treatment efficacy among the different forms of administration. The most commonly reported adverse events were nausea and vomiting (17.2%), followed by sepsis (14.1%) and metabolic acidosis (7.8%). However, this review was unable to assess the efficacy of sodium thiosulfate used alone, as some patients were under associated interventions. Moreover, the lack of a control group and the retrospective nature of the studies prevent definitive conclusions about the medication efficacy.^43^

There are reports of bisphosphonates used in patients with uremic calciphylaxis. A prospective case series with 11 patients showed there was a delay in calciphylaxis progression in all patients, two to four weeks after starting treatment; however, more controlled studies are required to establish its benefit.^44^

In patients with a confirmed or highly probable diagnosis, the therapeutic approaches should be implemented early due to the high morbidity and mortality rate of the disease.^3^, ^27^, ^28^

## Martorell’s hypertensive ischemic ulcer

### Definition

Hypertensive ulcers, also known as Martorell's ulcers, were first described by Martorell, Hines, and Farber in the 1940s.^45^ In 1945, Martorell published a case report describing supramalleolar ulcers due to arteriolitis associated with severe arterial hypertension in four obese women. From 1946 to 1947, Farber and Hines described the histopathological changes and defined the term “hypertensive ischemic leg ulcer”.^46^

By its initial definition, hypertensive ischemic ulcers (HIUs) occur in the presence of systemic arterial hypertension, usually long-standing and severe, in the absence of peripheral arterial occlusive disease or venous disease in the lower limbs, although more recently an association with these two comorbidities has been described.^47^

It is characterized by a rapidly progressive, extremely painful ulcer, which is often resistant to the usual topical treatments. HIUs should not be confused with arterial ulcers, since hypertensive ulcers cause changes in the microcirculation without impairing the macrocirculation, and ischemic arterial ulcers involve both micro and macrocirculation.^48^

### Epidemiology and risk factors

HIU is an uncommon cause of chronic ulcers of the lower limbs, although it is an underestimated disease, and it is difficult to find records of its prevalence.^32^ It accounts for 3% to 4% of the causes of chronic ulcers in the lower limbs, with a prevalence of 0.5% to 1% and an incidence of four to six cases per 1000 inhabitants/year.^32^

It is more common in women, especially after the age of 50, and only affects the lower limbs. It is important to rule out the diagnoses of peripheral venous and arterial occlusive disease in the lower limbs, which may indicate another etiology for the ulcers.

The associated risk factors include arterial hypertension in 100% of cases and type 2 diabetes mellitus in approximately 60% of the patients.^32^, ^45^, ^48^, ^49^

### Pathophysiology

Arterial hypertension is a systemic disease, causing widespread vascular alterations, such as macro and microangiopathy.^50^, ^51^ Long-term hypertension with or without diabetes mellitus induces the typical ischemic arteriosclerosis of small subcutaneous vessels. Calcification and hypertrophy of the intima layer of subcutaneous arterioles lead to increased vascular resistance, decreased tissue perfusion and local ischemia, and hypertensive ulcers can be considered target organ lesions. There is also an interruption of the physiological vasodilatory reflex in the distal arterioles of the obstructed region, further reducing tissue perfusion.^48^, ^52^ It is important to highlight the pathophysiological difference between HIU and arterial ischemic ulcer, with the latter resulting in a reduction in cutaneous perfusion pressure due to a reduction in arterial flow in the limb secondary to impairment in the macrocirculation, in addition to the effect in microcirculation.

### Clinical manifestations

**Fig. 6:**
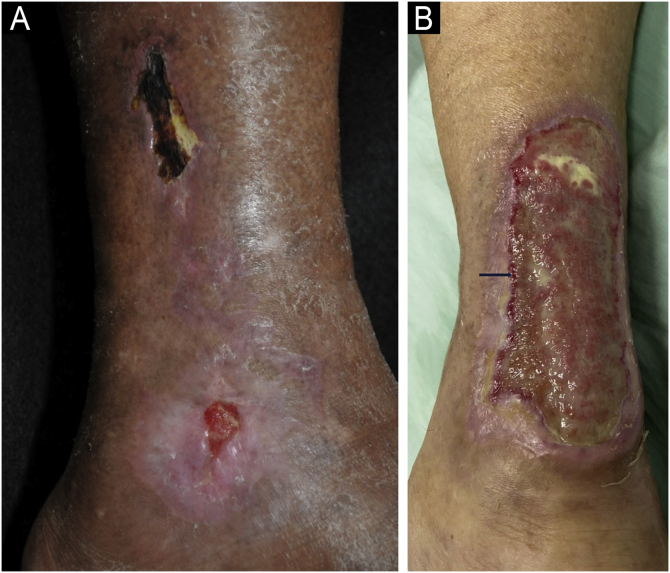
Hypertensive ischemic ulcers. (A) Ulcer with necrotic bed and purpuric edges on the lateral side of the leg and another smaller ulcer located on the lateral malleolus with good bed granulation tissue. (B) Extensive ulcer on the posterior region of the leg and calcaneus, with a devitalized bed and the ‘red lipstick sign’ (arrow) in the internal edge and erythematous-purpuric external edge.

The classic clinical manifestation comprises ulcers in the lower thirds of the legs, supramalleolar, and latero-posterior regions (Fig. 6A), unilateral or bilateral, which affect the Achilles tendon region (Fig. 6B).^45^, ^49^, ^52^ Approximately half of the affected individuals have bilateral manifestations during the course of the disease.^48^ Weber et al. performed a mapping of the location of HIU, finding a higher frequency in the medial third of the right leg, the lateral-posterior region followed by the lateral-anterior.^47^ The peri-ulcer region may become erythematous-purpuric; satellite lesions and livedo racemosa may occur.^45^, ^48^, ^49^ The ulcers are extremely painful, disproportionate to their size, and there is no improvement with changing the position of the leg,^32^, ^45^ such as elevation or hanging. Initially, the lesion appears as an erythematous plaque, which takes a violaceous color and then turns into a painful ulcer. It may show a necrotic appearance and bizarre contour^52^ (Fig. 6A). According to a recent publication describing the clinical characteristics of 69 patients with HIU, a new clinical sign, the “red lipstick sign” (reddish tissue around the inner edge of the ulcer – Fig. 6B), may aid in the clinical diagnosis associated with the purpuric edge and livedo racemosa.^53^

The pulses are usually palpable, and in most cases, there are no signs of peripheral arterial obstructive disease, with an ankle-brachial index (ABI) that is usually normal between 0.9 and 1.3; however, it is usually above 1.0 due to the increased vascular resistance caused by systemic arterial hypertension.^45^, ^54^ They are also characterized by occurring in patients with no signs of chronic venous insufficiency. However, the possibility of the patient having more than one of these conditions cannot be ruled out, and there may be concomitant peripheral arterial occlusive disease and chronic venous insufficiency, which makes diagnosis difficult.^52^ In the case series by Karppinen et al., 36.2% of patients with HIU had peripheral arterial disease and 18.8% had chronic venous insufficiency.^53^

### Diagnosis

Among the differential diagnoses are venous and arterial ulcers (more common than hypertensive ulcers), pyoderma gangrenosum (which also presents violaceous edges and necrotic bed),^55^ calciphylaxis (similar clinical and histopathological features).^52^ Pyoderma gangrenosum is the most commonly misdiagnosed condition in the early stages of HIU. One study reports that approximately 50% of patients with HIU were initially diagnosed with pyoderma gangrenosum.^48^

The diagnosis of HIU should be considered in patients with a history of severe arterial hypertension and can be challenging, considering that hypertensive etiology is less common than venous and arterial etiologies. Currently, there are no established diagnostic criteria, but comorbidities, severe pain, typical clinical features of the ulcers, the location and the histopathological findings guide the diagnosis. Histopathology can help in the differentiation; however, it the biopsy must be spindle-shaped and “deep” (including all levels of the skin up to the subcutaneous tissue and possibly fascia), and should be performed at the edge of the ulcer.^32^, ^49^

**Fig. 7:**
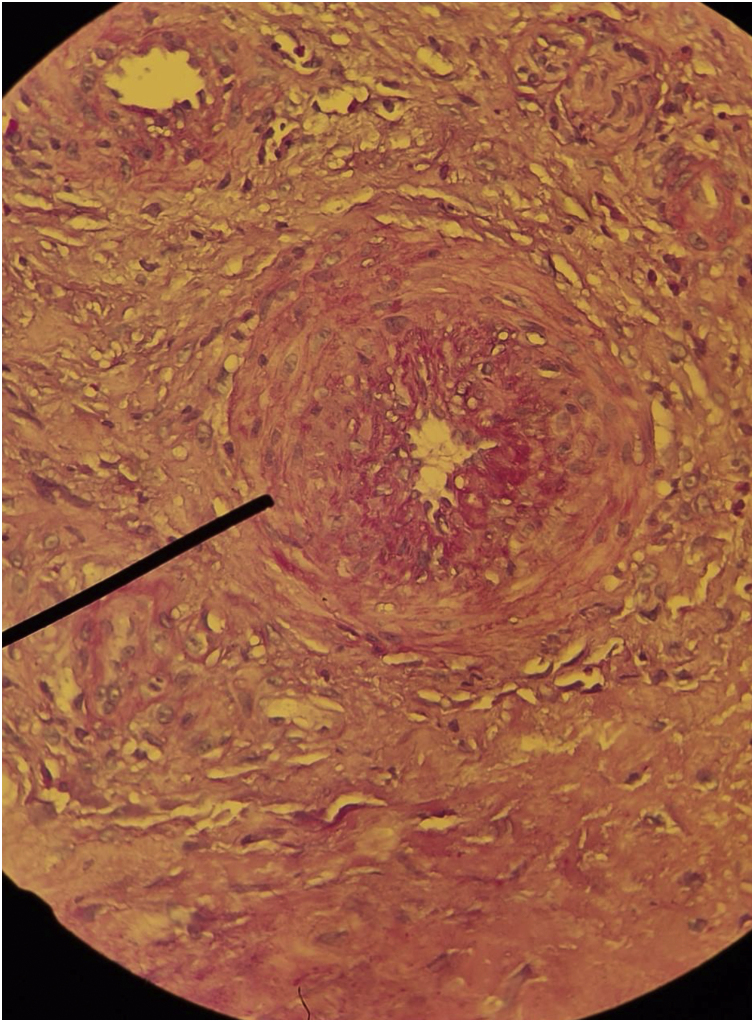
Histopathology of hypertensive ischemic ulcer. Concentric hypertrophy of the tunica media of an arteriole in the deep dermis (Hematoxylin & eosin, ×400).

The histopathology of HIU may reveal acanthosis, necrosis of the epidermis and dermis, as well as subcutaneous vessels with stenosis, calcification (medial calcinosis of Mönckeberg), medial layer hypertrophy, thrombosis and subendothelial hyalinosis, and alteration of the wall/lumen ratio (Fig. 7).^52^, ^56^ These histopathological alterations are also found in calciphylaxis,^3^ and the differentiation between HIU and calciphylaxis should be made by the history of difficult-to-control hypertension of many years, which favors hypertensive ulcers. A history of severe chronic kidney disease and altered serum calcium-phosphorus ratio favors the diagnosis of calciphylaxis.^3^, ^45^, ^48^, ^49^^,^^52^ On the other hand, there is spongiosis, dermal edema, hemosiderophages, fibrosis without necrosis, thrombosis, or hyalinosis in venous ulcers. In peripheral arterial occlusive disease there is a thin epidermis, dermal sclerosis, necrosis and thrombosis, without acanthosis or hyalinosis. Pyoderma gangrenosum, another important differential diagnosis, usually presents with extensive neutrophilic inflammatory infiltrate and there is no subcutaneous or medial calcification.^52^, ^56^ However, if the investigation of HIU the biopsy is very small, such as those performed by punch, there is a risk of only detecting characteristics similar to pyoderma gangrenosum, such as a neutrophilic infiltrate, and there is a high probability of missing the typical characteristics of HIU (subcutaneous stenotic arteriolosclerosis and medial calcification).^55^

### Treatment

The therapeutic approach should be multidisciplinary and consists of a combined regimen, including antihypertensive treatment, anticoagulation, pain control, surgical treatment and local measures directed at the ulcer.

Blood pressure control is always indicated; however, this is not capable of reversing the already established tissue alterations and effectively determine healing. Non-selective beta-blockers such as propranolol should be avoided or discontinued due to the worsening of peripheral vasoconstriction and consequently healing. Benefits have been reported with nifedipine (calcium channel blockers) and angiotensin-converting enzyme inhibitors (ACEIs).^57^, ^58^

Oral anticoagulants are indicated for anticoagulation, preferably those that are not vitamin K antagonists,^3^, ^32^ because the latter, which are widely used in oral anticoagulation, also inhibit a protein that protects against vitamin K-dependent calcification.^52^

Pain is difficult to control and may be of nociceptive and neuropathic origin. It is recommended, depending on the pain intensity, the use of common analgesics and opioids or anticonvulsants such as gabapentin or pregabalin. However, the first line of treatment for pain control is early surgical debridement and split-skin grafting.^54^ Punch grafting is a traditional and minimally invasive technique that has been associated with significant and rapid pain reduction in ulcers with different underlying causes, especially in HIU.^59^

Among local ulcer care, it is essential to manage devitalized tissues, maintain an ideal moisty environment, and control the bacterial load. Debridement of necrotic tissue can be performed using a superficial surgical technique. The use of becaplermin, a growth factor derived from human platelets and used for diabetic ulcers, has not shown improvement in pain or quality of life in relation to hydrogel, which favors autolytic debridement.^60^ Negative pressure therapy may aid the treatment.^57^

Compression therapy (25–30 mmHg) can be implemented once pain is controlled and provided there is no concomitant arterial disease, as it accelerates healing.

The comorbidities should be assessed and treated, such as diabetes mellitus and smoking. Other treatment indications include lumbar sympathectomy to control pain and blood pressure, and chlorpromazine or isoxsuprine to improve capillary flow.^45^, ^61^

## Final considerations

Livedoid vasculopathy, calciphylaxis and hypertensive ischemic ulcer are less common causes of chronic ulcers, but their identification is essential for the correct management of the affected patients. Table 4 summarizes the main risk factors, clinical manifestations, histopathology and treatment related to these three ischemic ulcers due to microcirculation impairment of the lower limbs, aiming to summarize what was presented in this article.

**Table 4:** Summary of the main risk factors, clinical manifestations, histopathology and treatment of livedoid vasculopathy, calciphylaxis and Martorell’s hypertensive ischemic ulcer.

Diagnosis	Main risk factors	Clinical manifestations	Histopathological findings	Treatment
**Livedoid vasculopathy**	Prothrombotic conditions	Small, deep, necrotic and intensely painful ulcers that affect the distal region of the legs, especially the malleolar region and dorsum of the foot.	Thrombi in the dermal vessels and sparse perivascular lymphocytic inflammatory infiltrate.	Control of risk factors and pain, local approach with dressings and debridement.
	Collagenoses			Drug therapy
	Sickle cell anemia			
	Infections (hepatitis B and C, HIV)	They heal slowly and leave characteristic atrophic ivory-white scars.	Deposition of fibrinoid material on the vessel walls	Hyperbaric oxygen therapy (see Table 2)
**Calciphylaxis**	Chronic kidney disease	Initially: painful, purpuric plaques or nodules associated with livedo racemosa.	Calcification of small vessels (von Kossa stain)	Control of risk factors, pain, local approach (debridement) with treatment of secondary infection, active treatment with sodium thiosulfate (see Table 3)
	Warfarin use	Progression to intensely painful, reticulated necrotic ulcers.	Intimal hyperplasia and thrombotic occlusion.	
	Obesity			
**Hypertensive ischemic ulcer**	Long-standing and difficult-to-control systemic arterial hypertension (SAH)	Initially: erythematous plaque that develops into a violaceous one, and then into a disproportionately painful ulcer.	Acanthosis, necrosis of the epidermis and dermis, stenosis of subcutaneous vessels with alteration of the wall/lumen ratio, calcification, thrombosis and subendothelial hyalinosis	Control of hypertension, analgesia, anticoagulation, local measures, surgical debridement and grafts
	Diabetes Mellitus	There may be associated necrosis, bizarre contour, and livedo racemosa.		

## Financial support

None declared.

## Authors' contributions

Priscila Neri Lacerda: Design and planning of the study; bibliographic survey, drafting and editing of the manuscript; critical review of the literature; critical review of the manuscript; approval of the final version of the manuscript.

Lucas Campos Garcia: Design and planning of the study; bibliographic survey, drafting and editing of the manuscript; critical review of the literature; critical review of the manuscript; approval of the final version of the manuscript.

Izabelle Ferreira da Silva Mazeto: Design and planning of the study; bibliographic survey, drafting and editing of the manuscript; critical review of the literature; critical review of the manuscript; approval of the final version of the manuscript.

Hélio Amante Miot: Interpretation of the histopathological tests, critical review of the manuscript; approval of the final version of the manuscript.

Luciana Patricia Fernandes Abbade: Design and planning of the study; bibliographic survey, drafting and editing of the manuscript; critical review of the literature; critical review of the manuscript; approval of the final version of the manuscript.

## Conflicts of interest

None declared.
